# Evaluation of sulphadoxine-pyrimethamine for intermittent preventive treatment of malaria in pregnancy: a retrospective birth outcomes study in Mansa, Zambia

**DOI:** 10.1186/s12936-015-0576-8

**Published:** 2015-02-07

**Authors:** Kimberly E Mace, Victor Chalwe, Bonnie L Katalenich, Michael Nambozi, Luamba Mubikayi, Chikuli K Mulele, Ryan E Wiegand, Scott J Filler, Mulakwa Kamuliwo, Allen S Craig, Kathrine R Tan

**Affiliations:** Malaria Branch, Division of Parasitic Diseases and Malaria, Center for Global Health, Centers for Disease Control and Prevention, Atlanta, GA USA; School of Medicine, Department of Public Health, University of Zambia, Lusaka, Zambia; United States Peace Corps, Lusaka, Zambia; Tropical Diseases Research Centre, Ndola, Zambia; Ndola Central Hospital, Ndola, Zambia; Wusakile Mine Hospital, Kitwe, Zambia; National Malaria Control Centre, Lusaka, Zambia

**Keywords:** *Plasmodium falciparum malaria*, Intermittent preventive treatment of malaria in pregnancy, Sulphadoxine-pyrimethamine, Zambia

## Abstract

**Background:**

Intermittent preventive treatment of malaria in pregnancy (IPTp) with sulphadoxine-pyrimethamine (SP) decreases placental parasitaemia, thus improving birth outcomes. Zambian policy recommends monthly SP-IPTp doses given presumptively during pregnancy at each antenatal examination, spaced one month apart after 16 weeks of gestation. The effectiveness of SP-IPTp was evaluated in Zambia where a recent study showed moderate prevalence of *Plasmodium falciparum* parasites with genetic mutations that confer SP resistance.

**Methods:**

HIV-negative women were enrolled at the time of delivery at two facilities in Mansa, Zambia, an area of high malaria transmission. Women were interviewed and SP exposure was determined by antenatal card documentation or self-reports. Using Poisson regression modelling, the effectiveness of SP-IPTp was evaluated for outcomes of parasitaemia (microscopic examination of maternal peripheral, cord, and placental blood films), maternal anaemia (Hb < 11 g/dl), placental infection (histopathology), and infant outcomes (low birth weight (LBW), preterm delivery, and small for gestational age) in women who took 0–4 doses of SP-IPTp.

**Results:**

Participants included 435 women, with a median age of 23 years (range 16–44). Thirty-four women took zero doses of SP-IPTp, while 115, 142 and 144 women took one, two, or ≥ three doses, respectively. Multivariate Poisson regression models considering age, mosquito net usage, indoor residual spraying, urban home, gravidity, facility, wet season delivery, and marital status showed that among paucigravid women ≥ two doses of SP-ITPp compared to one or less doses was associated with a protective effect on LBW (prevalence ratio (PR) 0.33, 95% confidence interval (CI) 0.12–0.91) and any infection (PR 0.76, CI 0.58–0.99). Multivariate models considering SP-IPTp as a continuous variable showed a protective dose–response association with LBW (paucigravid women: PR 0.54, CI 0.33–0.90, multigravid women: PR 0.63, CI 0.41–0.97).

**Conclusions:**

In Mansa, Zambia, an area of moderate SP resistance, ≥ two doses of SP-IPTp were associated with a protective effect from malaria in pregnancy, especially among paucigravid women. Each dose of SP-IPTp contributed to a 46 and 37% decrease in the frequency of LBW among paucigravid and multigravid women, respectively. SP-IPTp remains a viable strategy in this context.

## Background

Malaria during pregnancy is dangerous for women and infants. Approximately 32 million pregnancies per year occurring in regions of sub-Saharan Africa are at risk for malaria [1], and an estimated 200,000 of them occur in Zambia [2]. Malaria infection during pregnancy can result in adverse effects for both mother and infant, including severe maternal anaemia, low birth weight (LBW), and increased perinatal and infant mortality; adverse effects are more likely during a woman’s first or second pregnancy [3]. Women infected with HIV have a higher risk for adverse pregnancy outcomes [4], and the beneficial effects of SP-IPTp can be compromised by HIV status. Malaria is a substantial health concern in Zambia. In 2010, at the time of the study, the national prevalence of malaria parasitaemia in children under five years old was 16%; Luapula Province had the highest prevalence with 51% of children under five years infected [5]. The Zambian Ministry of Health recommends preventive measures to protect pregnant women from malaria including sleeping under an insecticide-treated mosquito net (ITN), and taking sulphadoxine-pyrimethamine (SP) regardless of malaria parasitaemia, after 16 weeks of gestation as intermittent preventive treatment in pregnancy (IPTp) with SP (SP-IPTp) [6]. At the time of the study, the Zambia guidelines recommended three doses of SP-IPTp, spaced one month apart in the second and third trimesters of pregnancy. Subsequently, Zambian guidelines were updated to be in line with the WHO 2012 recommendations for monthly dosing of SP-IPTp given at each antenatal visit in the second and third trimesters [7].

SP-IPTp protects from malaria during pregnancy, likely by clearing existing asymptomatic infections and by preventing new infections from occurring during the period after treatment, due to SP’s long half-life [8]. Earlier studies showed that SP-IPTp decreased placental malaria parasitaemia [9-17], maternal anaemia [11,12,15,18], and LBW [15-17,19]. Parasite resistance to SP threatens the effectiveness of SP-IPTp. Indeed malaria parasites developed SP resistance in Zambia [20] and in 2006 the adequate clinical and parasitological response for SP in children in Zambia was only 77% [21]. Other studies in sub-Saharan Africa suggest that SP-IPTp effectiveness is weakening [22,23]. The presence of *Plasmodium falciparum* dihydropteroate synthase (*dhps*) and dihydrofolate reductase (*dhfr*) mutations are associated with SP drug resistance in the *Plasmodium* parasite. The quintuple mutant haplotype, consisting of the N51I, C59R, S108N substitutions in *dhfr* and the A437G and K540E substitutions in *dhps*, has been associated with SP treatment failure [24]. A study conducted concurrently to the present study in Mansa, Zambia showed that among 84 specimens with complete haplotypes at *dhfr* loci 51, 59 and 108, and *dhps* loci 437 and 540, 61% had the quintuple mutant [25]. In comparison to other malaria regions, Zambia has intermediate frequencies of mutations that cause resistance to SP. A study conducted in Zambia showed that HIV-infected women require monthly dosing to obtain the benefits of SP-IPTp [26], however there is no comparable study for HIV-negative women in Zambia.

This study was designed in collaboration with the Malaria in Pregnancy (MiP) Consortium as part of a multi-site investigation into the effectiveness of SP-IPTp, where Zambia is one of eight sites in six countries, including Mali (two sites), Burkina Faso, Uganda, Kenya, and Malawi (two sites). Three modules were designed, including a therapeutic efficacy evaluation, determination of molecular markers of SP resistance and a retrospective cohort assessment of birth outcomes. This manuscript describes the Zambia birth outcomes component; the compiled analysis from the multi-site analysis and results from the other modules and sites are reported separately [25,27-30].

## Methods

### Study design and population

In Zambia, the retrospective-cohort birth outcomes study was conducted in Mansa, Luapula Province, at the Mansa General Hospital and Senama Rural Health Clinic. Both facilities serve the Mansa District population; Mansa General Hospital is a level-2 referral hospital with 326 beds and is located 0.5 km from the Mansa District Health Management Office (DHMO), and Senama Rural Health Clinic is situated 3 km from the DHMO, outside Mansa urban centre and has 20 beds [31]. The study period was from December 2009 to December 2010, spanning portions of the 2009–2010 and 2010–2011 rainy seasons, occurring generally from November to April. Women who presented for delivery at the participating facilities were enrolled by midwives trained in sample collection procedures related to this study. Inclusion criteria were: having an available SP-IPTp and HIV-test history recorded on an antenatal card, a singleton delivery, being ≥ 16 years old, and providing informed consent. Since HIV-positive women are recommended to take co-trimoxazole prophylaxis to prevent opportunistic infections, and SP-IPTp is not advised for pregnant women on co-trimoxazole due to toxicity concerns from simultaneous administration of the two drugs with similar mechanisms of action [8], only HIV-negative women were enrolled in this study. Women were excluded if they had ever had a positive HIV test result, if after the 16th gestational week they received either a blood transfusion or an anti-malarial medication other than SP-IPTp, if they delivered outside of the hospital, or if they lived outside the Mansa District. Despite the requirement to enrol only women who had an antenatal card, because stock-outs of SP occurred during the study period, it was possible to enrol women who did not take any SP-IPTp doses. Sometimes eligible women who birthed at the two facilities could not be screened and enrolled because a study-trained midwife was not on duty, or could not accommodate specimen and data collection procedures in addition to routine hospital responsibilities, or when study materials were stocked out. To minimize the likelihood of systematic selection bias, the study-trained midwives included those of both supervisor and non-supervisor status, and also included those that worked variable shifts, including during the day and night time.

### Parameters evaluated and outcome definitions

Enrolled women were interviewed using MiP Consortium-designed data collection instruments for the multi-site study, modified specifically for the Zambian context. Information collected included: demographics, ITN ownership and use, indoor residual spraying (IRS), and medical history. Antenatal cards served as the primary source of documented SP-IPTp dates. However, a small number of self-reported SP-doses, which were commonly associated with SP stock-outs at the antenatal clinics, were accepted. When possible, these doses were verified with receipts from purchase at pharmacies or from other medical records because women often filled the SP-IPTp prescription and retained the receipt with her ANC card. During data analysis, the dates of the non-antenatal clinic SP doses were validated to ensure they were not duplicated with those reported on the antenatal card. For all enrolled women, the study team of nurse midwives attempted to collect the following soon after delivery: haemoglobin (Hb) measurements, blood smears from maternal peripheral, placental and cord blood, placental specimens for histopathology, infant birth weight, gestational age at delivery assessed by Ballard score [32], and last menstrual period (LMP). Babies with a birth weight of ≤2,500 g were followed up at six weeks to assess mortality.

Moderate maternal anaemia was defined as Hb <11 g/dl; severe maternal anaemia was Hb <8 g/dl. Placental infection according to histopathology was classified according to the following guidelines: class 1: parasites present with no pigment in monocytes or fibrin; class 2: parasites present with pigment in monocytes +/− fibrin; class 3: parasites present with pigment in fibrin; class 4: no parasites, pigment only indicating past infection; class 5: no parasites or pigment present, indicating no infection [33]. Maternal, cord and placental blood smears were examined for presence of malaria parasites, plus parasite species and density for those positive. Infants weighing ≤2,500 g at birth were considered to be LBW; small for gestational age (SGA) was defined as being less than the tenth percentile in weight per gestational age determined by a longitudinal ultrasound-derived foetal nomogram for a sub-Saharan African population [34]. Preterm was defined as birth at <37 weeks of completed gestation. Finally, a ‘composite poor birth outcome’ was defined as any infant born with LBW, SGA or preterm.

### Laboratory methods

#### Malaria blood smears

Thick and thin smears were prepared from finger-stick blood samples following a standard protocol using a 5% Giemsa solution [35]. Trained, on-site technicians read thick blood smears to detect *Plasmodium* parasites and to calculate the parasite density according to the number of asexual parasites per 300 white blood cells (WBCs), assuming a total of 8,000 WBCs per μl. A thick blood smear was classified as negative if no asexual parasites were identified after 1,000 WBCs were counted. Thin blood smears were examined to determine *Plasmodium* species. For quality control, all slides were sent to a national-level laboratory and read a second time by a senior microscopist. If the results of the two readings were discrepant then the slide was read for a third time by a national-level, senior microscopist for a final determination.

Upon delivery, samples of placenta and placental blood were collected. An incision was made on the maternal side of the placenta and pooled blood was collected for thick smear examination as described for malaria blood smears.

#### Placental histopathology

Tissue samples from the maternal side of the placenta were collected; dimensions were approximately 2 cm × 2 cm in length and width, and 1 cm in depth. Specimens were immediately placed in buffered formalin-filled bottles and stored at room temperature. Within one month of collection, specimens were sent to Tropical Disease Research Centre (TDRC), Ndola, Zambia where tissues were wax embedded within two weeks, sectioned, stained with Gurr’s modified Giemsa or haematoxylin and eosin, and examined independently by two senior pathologists experienced in reading placental histopathology for malaria. For discrepant results, specimens were reviewed and consensus was determined. Placental histology was classified on a five-point scale as described by Rogerson *et al*. [33] and also characterized as active placental infection (class 1), chronic placental infections (classes 2 and 3), and past malaria placental infection (class 4).

### Sample size

Sample size was calculated for the main birth outcome of interest, evidence of placental malaria by histopathology. The major strata of interest for the primary independent variable, SP-IPTp doses, was ‘two or more doses of SP’ (reflecting full uptake of the WHO IPTp recommendations) *versus* ‘one or less doses of SP’; based on the 2008 Zambian National Malaria Indicator Survey, 66% of women surveyed received at least two doses of SP-IPTp [36]. It was also estimated that 20% of untreated women would have placental malaria and that treatment with at least two doses of SP-IPTp would result in 5% of treated women having placental malaria. To detect a decrease in placental malaria from 20 to 5% with 66% of women in the ‘two or more doses’ strata compared to one or less doses of SP-IPTp and with a 95% confidence level (CI) and a power of 0.8, 116 treated women and 60 untreated women were needed for a total of 176 women. To account for the design effect (DE = 2) of cluster sampling at the health facility level, this sample was doubled to 352. Accounting for possible losses due to problems with specimen quality, 10% was added to this amount for a total of 387 patients.

### Statistical analysis

Frequency counts and percentages were calculated for subject characteristics. The primary outcome was placental infection according to histopathology; secondary outcomes were LBW, preterm delivery, SGA, composite poor birth outcome, maternal anaemia, and any infection (defined as pathology class 1–4, OR blood smear positive (maternal, placental or cord blood)). The independent variable of interest was doses of SP-IPTp, evaluated for three categorical SP-IPTp variables: (i) < two *versus* ≥ two SP-IPTp doses, with the ≥ two SP-IPTp doses representing full uptake of the WHO IPTp recommendations for sub-Saharan African countries at the time of the study; (ii) < three *versus* ≥ three SP-IPTp doses, with ≥ three SP-IPTp doses reflecting full uptake of the Zambian national policy at the time; and, (iii) two *versus* ≥ three SP-IPTp doses to assess the added benefit between two doses and three or more doses. All analyses were performed in SAS version 9.3 (SAS Institute, Inc, Cary, NC, USA) and PROC GENMOD was used for all Poisson regression models with robust standard errors [37]. Bivariate, unadjusted prevalence ratios for the SP-IPTp effects were calculated and multivariate Poisson regression models were fit to adjust for cofactors. Cofactors considered for the models were: age group (<18, 18–35, >35), gravidity, sleeping under an ITN the last night at home, living in a home treated with IRS, living in an urban area, facility of enrolment, delivering during the wet season (November–April), marital status, and taking SP-IPTp doses outside of antenatal clinic visits. Cofactors included in the final adjusted models were selected based on strength of association in bivariate models (at the 0.1 level of significance for any outcome), biological plausibility, model fit statistics, and the number of outcome events. To assess if SP-IPTp exhibited a dose–response trend, SP-IPTp was defined as a continuous variable in unadjusted and multivariate models. Finally, Pearson’s Chi-square tests were employed to assess the effect of dose timing on outcomes. A two-sided p-value <0.05 was considered statistically significant.

### Human subjects review

Ethical approval for this study was obtained from institutional review boards at both the US Centers for Disease Control and Prevention and the Tropical Diseases Research Centre in Zambia. All participating women provided written informed consent.

## Results

**Figure 1:**
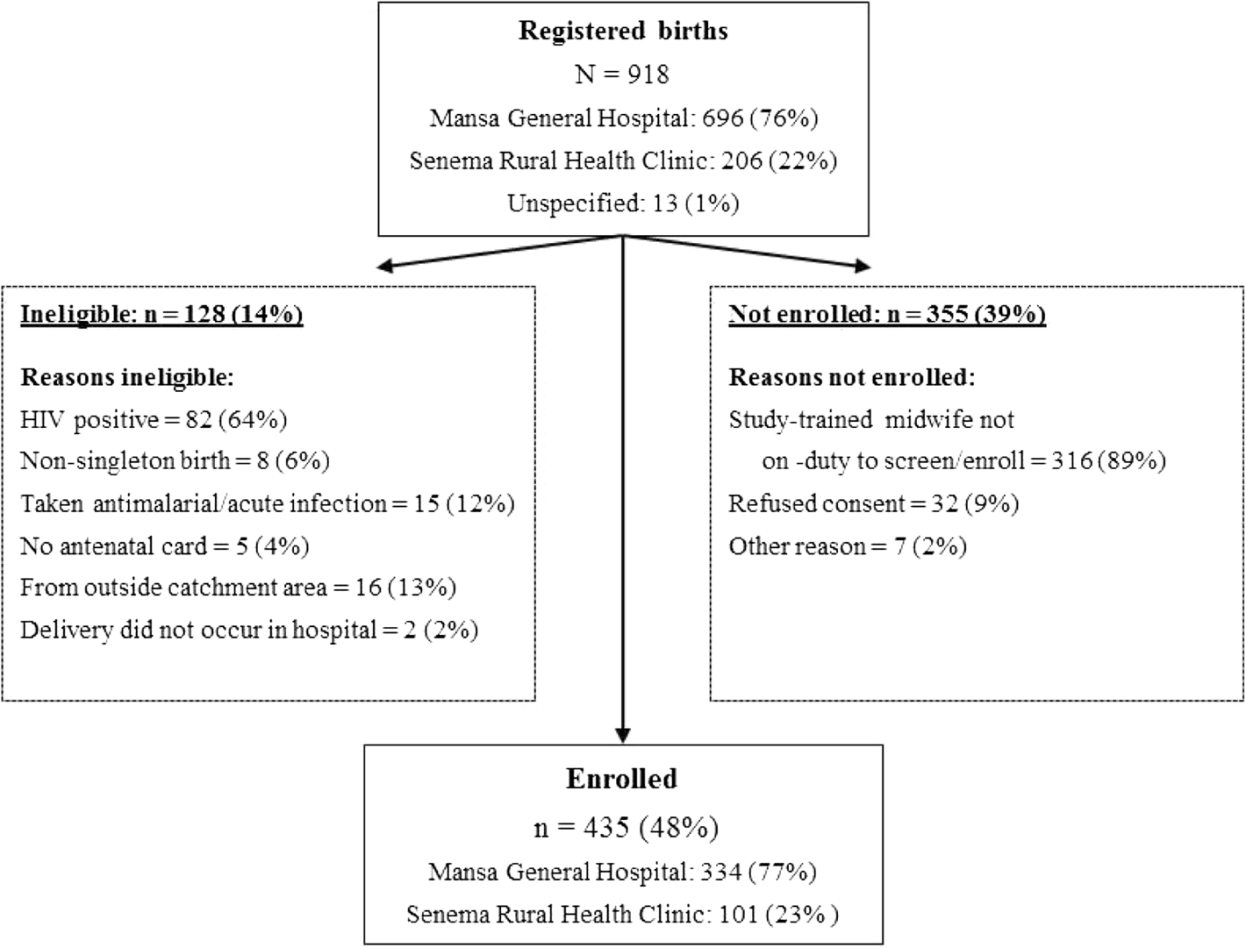
**Flow chart of registered births and participant enrolment.**

During the study period, a total of 918 births were registered at the two health facilities (Figure 1). Of these, 128 (14%) births were screened and found ineligible. Reasons for ineligibility included HIV positivity, non-singleton birth, having taken an anti-malarial for acute malaria infection, no antenatal card available (e.i., undocumented HIV status), being from outside the Mansa District, and for not delivering in one of the two hospitals. In addition 355 (39%) women were not enrolled because they refused consent, or there was no trained midwife on duty with sufficient time or study materials, or for other reasons. The most common reason that a birth was not screened for enrolment was because a trained midwife was not on duty, or was unable to conduct study procedures and still manage work priorities, 316 (89%). While all births at the two facilities were documented in the routine registers, demographic information was not available for the women not screened because a survey-trained midwife was not on duty. Table 1 shows the characteristics of the 435 women enrolled into the study. The median age was 23 years with a range from 16–44 years; 42 (10%) women were <18 years, and 33 (8%) were ≥35 years old. Thirty-seven per cent of women were pregnant for the first time (primigravid), and 51% were pregnant for the first or second time (paucigravid). Thirty-four (8%) women did not take any SP-IPTp during pregnancy; 115 (26%), 142 (33%) and 144 (33%) women took one, two or three + doses of SP-IPTp, respectively. In all, 435 enrolled women took 840 doses of SP-IPTp. Of these, 46 (5%) doses from 33 women were validated from self-report as being distinct from SP-IPTp doses recorded on the ANC card (13 additional doses were reported but could not be validated). Other subject characteristics are reported in Table 1.

**Table 1:** **Subject characteristics**

**Characteristic**	**n/N** ^**a**^ **(%)**
Aged <18 years old	42/432 (10)
Aged ≥35 years old	33/432 (8)
Doses of SP^b^-IPTp^c^ taken	
0 doses	34/435 (8)
1 dose	115/435 (26)
2 doses	142/435 (33)
3 doses	135/435 (31)
4 doses	9/435 (2)
Primigravid women	159/433 (37)
Paucigravid women	218/432 (51)
Multigravid women	214/432 (50)
From rural communities	255/433 (59)
Married	365/433 (84)
Home is treated with indoor residual spraying	149/411 (35)
Owns an insecticide treated net (ITN)	217/435 (50)
Slept under ITN during the last night at home	180/435 (42)
Delivered during the wet season	345/434 (80)
Self-reported doses of SP-IPTp	33/435 (8)

^a^Total contributing responses.

^b^SP = sulphadoxine pyrimethamine.

^c^IPTp = intermittent preventive treatment for malaria in pregnancy.

Infant and maternal outcomes are reported in Table 2. Of infants born in the cohort, 8% were LBW, 14% were preterm and 18% were SGA. There were a total of 133 (31%) infants born with a composite poor birth outcome (any of LBW, preterm or SGA). Of 31 LBW babies born alive, survivorship at six weeks was known for 28; 24 (86%) were alive at six weeks after birth. There were a total of 15 infant deaths in the study: nine were stillbirths (eight ≥28 weeks of gestation, one was <28 weeks of gestation), and six infants died immediately after, or within six weeks, of birth. No maternal deaths occurred among the cohort. At the time of delivery, more than one-third of all women had moderate anaemia, 157 (36%) women, while 14 (3%) women had severe anaemia.

**Table 2:** **Outcome frequencies**

**Outcome**	**n/N** ^**a**^ **(%)**
Placental infection by histopathology	
Abnormal (classes 1–4)	162/435 (37)
Active infection (class 1)	18/435 (4)
Chronic infection (classes 2 and 3)	32/435 (7)
Past infection (class 4)	112/435 (26)
Low birth weight (LBW) infant	34/435 (8)
Preterm infant	61/429 (14)
Small for gestational age (SGA) infant	76/428 (18)
Composite birth outcome (LBW, preterm, or SGA)	133/435 (31)
Stillbirth	9/432 (2)
Infant death within six weeks of birth	6/431 (1)
Maternal anaemia	
Moderate anaemia (≥8.0 Hb <11.0 g/dl)	157/435 (36)
Severe anaemia (Hb <8.0 g/dl)	14/435 (3)
Blood smear positive for malaria parasites	
Maternal peripheral	21/419 (5)
Cord blood	9/428 (2)
Placental	19/430 (4)
Any smear	28/420 (7)
Parasite density (parasites/ul); min, max, median	106.7, >200,000, 3333.3

^a^Number with outcome/total contributing responses.

Blood smear analysis showed that 21 (5%), nine (2%), and 19 (4%) had a positive *P. falciparum* maternal peripheral, cord and placental blood smear, respectively; no other malaria species were detected. Twenty-eight (7%) subjects had any positive blood smear. More than one-third of all placental histopathology specimens were abnormal for malaria infection 162 (37%), the majority of these being classified as past infection (class 4), 112 (26%). Placental histopathology identified 18 (4%) active infections (class 1), and 32 (7%) chronic infections (classes 2 and 3).

An unadjusted analysis examining the effects of SP exposure dichotomized in various ways and its effects on the outcomes of interest is summarized in Table 3. Two or more SP-IPTp doses among all women was associated with a protective effect for placental infection, LBW, preterm delivery, composite poor birth outcome, and any infection. Three doses of SP-IPTp compared to < three doses remained significantly associated with a reduction of LBW, preterm deliveries and composite poor birth outcome, for women of all gravidity. The difference between two and three or more SP-IPTp doses was not significantly different for any outcome or gravidity level.

**Table 3:** **Unadjusted model: SP** ^**a**^ **-IPTp** ^**b**^ **doses (categorical variables) associated with outcomes of interest**

**Unadjusted model**	**IPTp <2** ***vs*** **≥ 2 SP doses n, PR** ^**c**^ **(95% CI** ^**d**^ **)**	**IPTp <3** ***vs*** **≥3 SP doses n, PR (95% CI)**	**IPTp 2** ***vs*** **≥3 SP doses n, PR (95% CI)**
Placental infection^e^			
All gravidity	435, 0.78 (0.61-0.99)	435, 0.80 (0.61-1.06)	286, 0.89 (0.64-1.23)
Paucigravid	218, 0.67 (0.51-0.88)	218, 0.80 (0.57-1.12)	147, 0.98 (0.66-1.47)
Multigravid	214, 0.93 (0.60-1.44)	214, 0.81 (0.50-1.30)	137, 0.79 (0.46-1.36)
Low birth weight (<2,500 g)			
All gravidity	435, 0.41 (0.22-0.79)	435, 0.35 (0.14-0.88)	286, 0.49 (0.17-1.41)
Paucigravid	218, 0.27 (0.09-0.77)	218, 0.36 (0.08-1.56)	147, 0.75 (0.13-4.38)
Multigravid	214, 0.51 (0.21-1.19)	214, 0.23 (0.05-0.96)	137, 0.25 (0.05-1.16)
Preterm (<37 weeks)			
All gravidity	429, 0.51 (0.32-0.81)	429, 0.40 (0.21-0.77)	281, 0.50 (0.24-1.02)
Paucigravid	216, 0.81 (0.43-1.51)	216, 0.64 (0.31-1.34)	146, 0.66 (0.29-1.47)
Multigravid	210, 0.26 (0.12-0.56)	210, 0.16 (0.04-0.67)	133, 0.29 (0.06-1.39)
Small for gestational age			
All gravidity	428, 0.95 (0.62-1.45)	428, 0.83 (0.53-1.30)	281, 0.81 (0.48-1.35)
Paucigravid	216, 1.11 (0.62-1.99)	216, 0.94 (0.53-1.69)	146, 0.88 (0.46-1.67)
Multigravid	209, 0.79 (0.41-1.52)	209, 0.68 (0.32-1.43)	133, 0.70 (0.29-1.66)
Composite birth outcome^f^			
All gravidity	435, 0.74 (0.56-0.98)	435, 0.64 (0.45-0.90)	286, 0.69 (0.47-1.01)
Paucigravid	218, 0.99 (0.67-1.45)	218, 0.82 (0.55-1.25)	147, 0.79 (0.50-1.25)
Multigravid	214, 0.50 (0.32-0.79)	214, 0.43 (0.23-0.80)	137, 0.55 (0.27-1.12)
Anemia (Hb <11 g/dl)			
All gravidity	434, 0.89 (0.70-1.13)	434, 0.87 (0.67-1.13)	285, 0.90 (0.67-1.22)
Paucigravid	217, 0.75 (0.55-1.03)	217, 0.72 (0.49-1.06)	146, 0.79 (0.51-1.22)
Multigravid	214, 1.06 (0.74-1.53)	214, 1.08 (0.75-1.54)	137, 1.06 (0.69-1.62)
Any infection^g^			
All gravidity	435, 0.78 (0.61-0.99)	435, 0.79 (0.60-1.04)	286, 0.87 (0.64-1.20)
Paucigravid	218, 0.66 (0.50-0.86)	218, 0.80 (0.57-1.10)	147, 0.99 (0.67-1.46)
Multigravid	214, 0.95 (0.62-1.47)	214, 0.79 (0.49-1.26)	137, 0.75 (0.44-1.28)

^a^SP = sulphadoxine pyrimethamine.

^b^IPTp = intermittent preventive treatment for malaria in pregnancy.

^c^PR = prevalence ratio.

^d^Denotes 95% confidence interval.

^e^Placental infection outcome: classes 1–4 *vs* class 5.

^f^Composite birth outcome defined as infants born with any of: LBW, SGA, or preterm.

^g^Any infection defined as: pathology classes 1–4, blood smear positive (maternal, placental or cord blood).

Outcomes also varied by gravidity (Table 3). Paucigravid women were more likely to be associated with protection from LBW, placental histopathology malaria abnormalities, or any infection. In contrast, multigravid women were more likely to be protected from preterm delivery, and composite poor birth outcome. In addition, among multigravid women, protection from LBW was not associated with two doses of SP-IPTp, but was observed with three or more SP-IPTp doses.

Outcomes significant in the unadjusted analysis were assessed with Poisson multivariate regression models, including gravidity as an interaction term with SP-IPTp, and age group (<18, 18–35, >35), sleeping under an ITN the previous night at home, living in a home that is IRS treated, living in an urban area, facility of enrolment, delivering during the wet season (November–April), and marital status (Table 4). Self-reported SP-IPTp doses were considered and tested as a cofactor, but were not found to be significant (at a 0.1 level) for any outcome of interest. All three SP-IPTp dosing comparisons were assessed, although for some outcomes, SP-IPTp two *versus* ≥ three doses could not be computed because of an inadequate number of responses. In paucigravid women two or more SP-IPTp doses were protective for LBW and any infection. Among multigravid women, ≥two and ≥ three SP-IPTp doses were associated with fewer preterm deliveries and composite poor birth outcomes. Three or more doses of SP-IPTp compared to two doses were not significant for either of the outcomes for which it could be computed (placental infection, and any infection).

**Table 4:** **Multivariate regression: SP** ^**a**^ **-IPTp** ^**b**^ **doses (categorical variables) associated with outcomes of interest**

**Adjusted model** ^**c**^	**<2** ***vs*** **≥2 doses PR** ^**d**^ **(95% CI** ^**e**^ **)**	**<3** ***vs*** **≥ 3 doses PR (95% CI)**	**2** ***vs*** **≥3 doses PR (95% CI)**
Placental infection^f^			
Paucigravid	0.77 (0.58- 1.01)	0.89 (0.64-1.23)	1.06 (0.71-1.57)
Multigravid	1.00 (0.66-1.52)	0.87 (0.55-1.39)	0.83 (0.49-1.39)
Low birth weight (<2,500 g)			
Paucigravid	0.33 (0.12-0.91)	0.43 (0.10-1.81)	Could not compute
Multigravid	0.51 (0.21-1.25)	0.26 (0.06-1.11)	Could not compute
Preterm (<37 weeks)			
Paucigravid	0.93 (0.49-1.78)	0.70 (0.34-1.44)	Could not compute
Multigravid	0.28 (0.13-0.60)	0.15 (0.04-0.55)	Could not compute
Composite birth outcome^g^			
Paucigravid	1.09 (0.73-1.61)	0.84 (0.54-1.31)	Could not compute
Multigravid	0.50 (0.32-0.78)	0.43 (0.24-0.80)	Could not compute
Any infection^h^			
Paucigravid	0.76 (0.58-0.99)	0.88 (0.65-1.21)	1.06 (0.72-1.55)
Multigravid	1.04 (0.69-1.58)	0.86 (0.54-1.36)	0.79 (0.48-1.33)

^a^SP = sulphadoxine pyrimethamine.

^b^IPTp = intermittent preventive treatment for malaria in pregnancy.

^c^ Model adjusted for: Age group (<18, 18–35, >35), gravidity as an interaction term, sleeping under an ITN the last night at home, living in a home treated with IRS, urban, facility, delivered during the wet season, marital status.

^d^PR = prevalence ratio.

^e^CI = 95% confidence interval.

^f^Placental infection outcome: histopathology classes 1–4 *vs* class 5.

^g^Composite birth outcome defined as infants born with any of: LBW, SGA or preterm.

^h^Any infection defined as: pathology classes 1–4, blood smear positive (maternal, placental or cord blood).

Table 5 summarizes the results of an unadjusted assessment of the dose–response effect of SP-IPTp on outcomes, considering SP-doses as a continuous variable, and the interaction of gravidity and number of SP-IPTp doses taken into account. Among all women, the trend p-values was significant (<0.05) for outcomes of placental infection by histopathology, LBW, preterm delivery, composite poor birth outcome, and any infection.

**Table 5:** **Unadjusted trend analysis: SP** ^**a**^ **-IPTp** ^**b**^ **doses (continuous variable) associated with outcomes of interest**

**Outcome**	**Trend prevalence ratio estimate (95% CI** ^**c**^ **)**	**Trend p-value**
Placental infection^d^		
All gravidity	0.87 (0.77-0.99)	0.03
Paucigravid	0.84 (0.72-0.97)	0.02
Multigravid	0.91 (0.73-1.12)	0.37
Low birth weight (<2,500 g)		
All gravidity	0.59 (0.43-0.81)	<0.01
Paucigravid	0.50 (0.29-0.85)	0.01
Multigravid	0.62 (0.42-0.90)	0.01
Preterm (<37 weeks)		
All gravidity	0.65 (0.51-0.81)	<0.01
Paucigravid	0.82 (0.60-1.12)	0.21
Multigravid	0.47 (0.33-0.66)	<0.01
Small for gestational age		
All gravidity	0.93 (0.75-1.15)	0.49
Paucigravid	0.99 (0.74-1.32)	0.94
Multigravid	0.85 (0.62-1.17)	0.32
Composite birth outcome^e^		
All gravidity	0.80 (0.70-0.92)	<0.01
Paucigravid	0.92 (0.76-1.11)	0.37
Multigravid	0.67 (0.54-0.84)	<0.01
Anemia (Hb <11 g/dl)		
All gravidity	0.93 (0.82-1.05)	0.24
Paucigravid	0.84 (0.72-0.99)	0.04
Multigravid	1.03 (0.86-1.23)	0.74
Any infection^f^		
All gravidity	0.87 (0.77-0.98)	0.03
Paucigravid	0.84 (0.72-0.96)	0.01
Multigravid	0.91 (0.74-1.12)	0.38

^a^SP = sulphadoxine pyrimethamine.

^b^IPTp = intermittent preventive treatment for malaria in pregnancy.

^c^CI = 95% confidence interval.

^d^Placental infection outcome: histopathology classes 1–4 *vs* class 5.

^e^Composite birth outcome defined as infants born with any of: LBW, SGA, or preterm.

^f^Any infection defined as pathology classes 1–4, blood smear positive (maternal, placental or cord blood).

Multivariate Poisson regression analysis with the same covariates as previously mentioned (age group (<18, 18–35, >35), sleeping under an ITN the previous night at home, living in a home that is IRS treated, living in an urban area, facility of enrolment, delivering during the wet season, and marital status) shows that protection from LBW was significantly associated with increased SP-IPTp doses for both paucigravid and multigravid women (Table 6). For each dose of SP-IPTp there was a 46 and 37% decrease in the frequency of LBW among paucigravid and multigravid women, respectively. Placental infection by histopathology and any infection were not significantly associated with SP-IPTp doses in this analysis. Among multigravid women, the dose–response effect from SP-IPTp remained significantly associated with protection from preterm delivery and the composite poor birth outcome.

**Table 6:** **Multivariate logistic regression trend analysis: SP** ^**a**^ **-IPTp** ^**b**^ **doses (continuous variable) associated with outcomes of interest**

**Adjusted model** ^**c**^	**Trend prevalence ratio estimate (95% CI** ^**d**^ **)**	**Trend p-value**
Placental infection^e^		
Paucigravid	0.88 (0.77-1.04)	0.13
Multigravid	0.94 (0.76-1.16)	0.58
Low birth weight (<2,500 g)		
Paucigravid	0.54 (0.33-0.90)	0.02
Multigravid	0.63 (0.41-0.97)	0.04
Preterm (<37 weeks)		
Paucigravid	0.85 (0.62-1.17)	0.32
Multigravid	0.48 (0.34-0.69)	<0.0001
Composite birth outcome^f^		
Paucigravid	0.94 (0.77-1.15)	0.56
Multigravid	0.66 (0.53-0.82)	<0.001
Any infection^g^		
Paucigravid	0.89 (0.77-1.03)	0.13
Multigravid	0.95 (0.77-1.17)	0.63

^a^SP = sulphadoxine pyrimethamine.

^b^IPTp = intermittent preventive treatment for malaria in pregnancy.

^c^Model adjusts for: Age group (<18, 18–35, >35), gravidity as an interaction term, sleeping under an ITN the last night at home, living in a home treated with IRS, urban, facility, delivered during the wet season, marital status.

^d^CI = 95% confidence interval.

^e^Placental infection outcome: histopathology classes 1–4 *vs* class 5.

^f^Composite birth outcome defined as infants born with any of: LBW, SGA, or preterm.

^g^Any infection defined as pathology classes 1–4, blood smear positive (maternal, placental or cord blood).

The effect of the timing of the SP-IPTp doses on outcomes was assessed among 392 women who had taken ≥ one SP-IPTp dose, and for which the complete date of the SP-IPTp dose was known. A total of 172 (44%) women took their last dose of SP-IPTp within the four weeks prior to birth. The only outcome that was significantly associated with the timing of the last SP-IPTp dose (Pearson’s Chi-square test) was parasite positivity (parasites found on any blood smear, or histopathology classes 1–3); 16.8% of women were parasite positive among those who did not take SP-IPTp during the four weeks prior to birth *versus* 8.7% positive among those who did take SP-IPTp during the four weeks prior to birth (p = 0.02). There was no effect of the timing of SP-IPTp on the following outcomes: placental infection, moderate anaemia, LBW, pre-term delivery, SGA, composite poor birth outcome, and any infection.

## Discussion

This study found that SP-IPTp in Mansa, Zambia protected pregnant women from poor birth outcomes, and that the degree and type of protection afforded by SP-IPTp was dependent on the specific outcome, gravidity, and number of SP-IPTp doses taken. Two or more doses of SP-IPTp were determined to be a threshold associated with protection of paucigravid women from outcomes of placental malaria, LBW, and any malaria infection. Likewise, at least two or more doses of SP-IPTp were associated with protection of multigravid women from preterm delivery and composite poor birth outcome. In addition, analysis of SP-IPTp as a continuous variable, to assess the trend-effect for each subsequent dose, showed that each dose of SP-IPTp confers a protective benefit for LBW among women of any gravidity (Table 6). Furthermore, the setting in which these outcomes were observed was described in an SP-IPTp therapeutic efficacy study, conducted in parallel to this one, in Mansa, Zambia, showing that among asymptomatic, parasitaemic, HIV-negative women enrolled in their second or third trimester, SP-IPTp had a 26% failure rate in terms of recurrent parasitaemia, and found an intermediate prevalence of genetic mutations that contribute to SP resistance [25].

This work provides valuable information on the continued effectiveness of SP-IPTp in transmission settings that have moderate prevalence of *dhps* and *dhfr* mutant alleles. Previous studies have examined SP-IPTp outcomes in areas of high prevalence of the quintuple SP mutations [22,38-42], sometimes demonstrating that protective effectiveness is waning or SP-IPTp is harmful [22,43]. Alternatively, in the West Africa region, while SP mutations are not uncommon, there is lower prevalence of the quintuple mutation [40,44] and continued effectiveness of SP-IPTp is likely to remain high [45,46].

In 2012 WHO clarified SP-IPTp recommendations for areas of moderate-to high transmission, continuing the recommendation that women should receive four antenatal care visits spaced at least one month apart in the second and third trimester, but specifying that a dose of SP-IPTp should be administered at each visit [7]; Zambia adopted a policy aligned with the current WHO recommendations. Results presented herein support the current SP-IPTp policies and provide further evidence that pregnant women continue to benefit from three or more doses of SP-IPTp, by showing that SP-IPTp had a protective dose–response effect among all women for the outcome of LBW (Table 6). Operationally, the clarified guidelines should be straightforward for antenatal clinics to implement, increasing the likelihood that women will benefit from an increased number of SP-IPTp doses. To improve SP-IPTp uptake, measures such as supervision of healthcare workers to improve provision of SP-IPTp, improvement of supply chain management to prevent SP stock-outs, and text messages serving as antenatal care reminders, could be considered.

Results herein illustrate differences in consideration of outcomes and gravidity. For the primary outcome of placental infection, at least two doses of SP-IPTp are protective for paucigravid women and not multigravid women. In addition, paucigravid women were similarly protected from LBW and any infection, in contrast to multigravid women. The outcomes where SP-IPTp is significantly protective only among multigravid women are preterm delivery and the composite poor birth outcome (which includes, and is driven by preterm delivery) (Table 4). While there is not a single compelling reason to explain this gravidity difference for the preterm outcome, the following explanations are plausible: (i) among primigravid women, it is possible that there was not sufficient power to detect a protective effect against preterm delivery, (ii) preterm is assessed using last menstrual period and Ballard scores, methodologies which can be subject to error, and could have resulted in misclassification bias. These results support the conclusion that SP-IPTp confers a protective effect among women of any gravidity status, although the protection is stronger among paucigravid women for the outcomes of LBW, placental infection, and any infection.

This study has the following limitations: it was designed to be observational where women were not randomized to receive different amounts of SP-IPTp doses. While relatively equal distribution of SP-IPTp for one, two or three or more doses (115, 142, and 144 women, respectively) was observed, only 34 women received zero doses of SP-IPTp, limiting the ability to analyse the effectiveness of any SP-IPTp doses compared to the group of women who received none. It is possible that there is misclassification of some SP-IPTp doses, especially for those 46 doses not recorded on ANC cards. Treating SP-IPTp dosage as a continuous variable in modelling may mask potential non-linear effects. It was not possible to determine the quality of the SP doses provided at antenatal clinics across the Mansa District area. However, the dose-timing analysis found that taking SP-IPTp in the four weeks prior to birth was associated with a decrease in parasite positivity, suggesting generally that the administered SP had anti-parasitic properties. One-third of all births registered during the study period at the two facilities were not screened for enrolment, and since demographic information was not available for the non-screened women it is impossible to evaluate whether women enrolled were representative of the true population of women giving birth at the two sites. Although all enrolled women had an HIV-negative test result recorded during their pregnancy, it is possible that HIV-positive women were enrolled; however the effect of this error would have biased results towards the null hypothesis. Additionally, it was not possible to control for antenatal visits, and this could affect the study outcomes, including birth weight [47].

## Conclusions

In Mansa, Zambia, an area of moderate SP resistance, two or more doses of SP-IPTp are associated with a protective effect from malaria in pregnancy, especially among paucigravid women. Each dose of SP-IPTp contributed to a 46 and 37% decrease in the frequency of LBW among paucigravid and multigravid women, respectively. The results herein suggest that SP-IPTp confers protective benefits to pregnant women and their infants in this transmission setting, despite a previously described intermediate prevalence of genetic mutations that contribute to SP resistance; the SP-IPTp strategy should continue to be supported. To protect women from deleterious effects of malaria during pregnancy, update of SP-IPTp should be improved; SP supply chain management, implementing text message antenatal reminders, and supervision of healthcare providers could be considered to ensure women have optimal access to SP-IPTp.
